# The clinical efficacy of anterior cervical discectomy and fusion with ROI-C device vs. plate-cage in managing traumatic central cord syndrome

**DOI:** 10.3389/fsurg.2022.1055317

**Published:** 2023-01-06

**Authors:** Dawei Song, Zicheng Deng, Tao Feng, Jinning Wang, Yijie Liu, Heng Wang, Huilin Yang, Junjie Niu

**Affiliations:** Department of Orthopaedics, The First Affiliated Hospital of Soochow University, Suzhou, China

**Keywords:** traumatic central cord syndrome, anterior cervical discectomy and fusion, ROI-C device, plate-cage system, ASIA impairment scale, dysphagia

## Abstract

**Purpose:**

To assess the efficacy and complications of anterior cervical discectomy and fusion (ACDF) with ROI-C device vs. conventional anterior plate and cage system (APCS) in managing traumatic central cord syndrome (TCCS).

**Methods:**

A total of 37 patients diagnosed with TCCS who underwent ACDF with ROI-C implant and APCS were recruited in this retrospective study from June 2012 to February 2020. Radiological parameters and clinical results were recorded and compared through follow-up time. Characteristics of patients and complications were also recorded.

**Results:**

All patients tolerated the procedure well. The average follow-up time was 25.00 ± 7.99 months in the ROI-C group, and 21.29 ± 7.41 months in the APCS group. The blood loss and operation time were significantly lower in the ROI-C group than in the APCS group. Radiological parameters and clinical results were all improved postoperatively and maintained at the final follow-up. Fusion was achieved in all patients. ROI-C group had a lower incidence of postoperative dysphagia than the APCS group. Only 1 case of ALD was observed at the final follow-up in the APCS group.

**Conclusions:**

Both ROI-C device and APCS demonstrated satisfactory clinical effects and safety in managing symptomatic single-level traumatic central cord syndrome with underlying instability. Both techniques could improve and maintain cervical lordosis and disc height. ROI-C device was related to a lower incidence of postoperative dysphagia, shorter operation time, and less blood loss.

## Introduction

Regarding the most common type of incomplete spinal cord injury (SCI), traumatic central cord syndrome (TCCS) is often associated with hyperextension trauma, leading to disproportionately more damage to the upper than in the lower extremities, bladder dysfunction, and variable sensory loss below the level of injury (1). After first proposed by Schneider et al. in 1954 (2), conservative treatment was regarded as a reliable method in managing TCCS for mild TCCS with slight neurological impairment (3). However, for patients with TCCS, there is a great probability of a combination of preexisted cervical spinal cord compression due to degeneration, leading to correlated symptoms and further segmental instability. With the development of cervical spinal surgery and the recognition of the injury mechanism, operation is becoming an effective and secure way of treating TCCS, especially with symptomatic spinal compression and segmental instability.

Determined by angular displacement and vertebral body translation, cervical spinal instability is the key indication for surgery, which needs reconstruction of both alignment and stability (4). Nevertheless, dynamic x-ray was not suitable for patients with trauma in case of aggravation of symptoms. Besides, prevertebral edema signals from magnetic resonance (MR) and lesions from intervertebral space found during operation are potential factors of spinal instability (5).

For patients with instability from the anterior column, anterior cervical discectomy and fusion (ACDF) can achieve direct decompression of protrusions, restore intervertebral height, correct cervical alignment, and attain solid fusion. A stand-alone cage has less stability to obtain solid fusion (6). Anterior plating and cage system (APCS) was introduced thereafter to further stabilize the cervical spine in ACDF (7). However, relative complications were recognized as plate and screw fracture, malposition, loosening, dysphagia and future degeneration of adjacent levels (8–12).

The ROI-C peek cage device, a type of zero-profile anchored cage (ZPAC) implant without using anterior plating, has been developed as it can further increase cervical stability through 2 integrated self-locking clips compared with stand-alone cages (9, 10, 13). Currently, there is only few clinical research about the ROI-C device, and few studies concentrated on its application in TCCS. In the present study, we perform this retrospective clinical research to assess the efficacy of ACDF with ROI-C device vs. APCS in managing single-level symptomatic TCCS with potential instability.

## Materials and methods

### Characteristics of patients

This study was approved by the Institutional Ethics Committee of our institution. Informed consent was obtained from all individual participants included in the study. A total of 37 patients diagnosed with TCCS with potential instability underwent single-level ACDF with ROI-C implant (LDR, Troyes, France) and APCS (Medtronic, USA) were recruited in this retrospective study from June 2012 to February 2020. Patients' characteristics were summarized in Table 1. Data were collected and analyzed at admission preoperatively, postoperative at discharge, 3 months postoperatively, and at final follow-up time.

**Table 1:** Characteristics of patients.

Characteristics	ROI-C	APCS
Number	20	17
Age (years)	51.90 ± 9.64	50.00 ± 9.37
Gender (male/female)	17/3	13/4
Follow-up time (months)	25.00 ± 7.99	21.29 ± 7.41
Inpatient days	13.10 ± 4.18	13.24 ± 2.80
Time before surgery (days)	5.35 ± 2.52	5.59 ± 2.12
Causes for trauma		
Falling	11	8
Traffic accident	6	7
Sports	3	2
Levels operated	20	17
C3–4	8 (40.0%)	2 (11.8%)
C4–5	6 (30.0%)	5 (29.4%)
C5–6	6 (30.0%)	8 (47.1%)
C6–7	0 (0.0%)	2 (11.7%)
Operation time (minutes)	89.40 ± 14.03*	110.29 ± 12.31*
Intraoperative blood loss (ml)	58.50 ± 7.72*	93.53 ± 15.18*

APCS, Anterior plating and cage system.

*Statistical significance achieved compared between groups (*P* < 0.05).

The inclusion criteria were: (1) incomplete single-level spinal cord damage symptom due to related trauma; (2) identified spinal cord compression sign, prevertebral edema signal from magnetic resonance, image (MRI) or lesion from intervertebral space found during operation; (3) no cervical vertebral fracture or dislocation. Exclusion criteria were: (1) severe brain damage, pre-traumatic neurological paralysis symptoms, and complete spinal cord injury; (2) history of cervical spine surgery; (3) multi-level spinal cord injury, cervical bony fracture, evident dislocation, subluxation, ossification of the posterior longitudinal ligament, infection, tumor, and severe osteoporosis; (4) severe spinal canal stenosis.

### Surgical procedure

After general anesthesia, patients were placed in supine position. An anterior Smith–Robinson approach was applied for exposure and distraction of intervertebral space. Intervertebral discs and endplate cartilage was removed without excessive scraping of the subchondral bone for preparation of arthrodesis and prevention of cage subsidence. The posterior longitudinal ligament was excised for further decompression of protrusions. For the ROI-C group, an appropriate-sized ROI-C implant was inserted into the intervertebral space monitored by fluoroscopy. After removal of the Caspar distracter, 2 anchoring clips were then installed in the up and lower vertebral body to achieve solid stabilization. For the plate and cage group, a suitable-sized cage and plate were placed, and self-tapping screws were fixed through the plate to the vertebrae.

### Radiological evaluation

**Figure 1:**
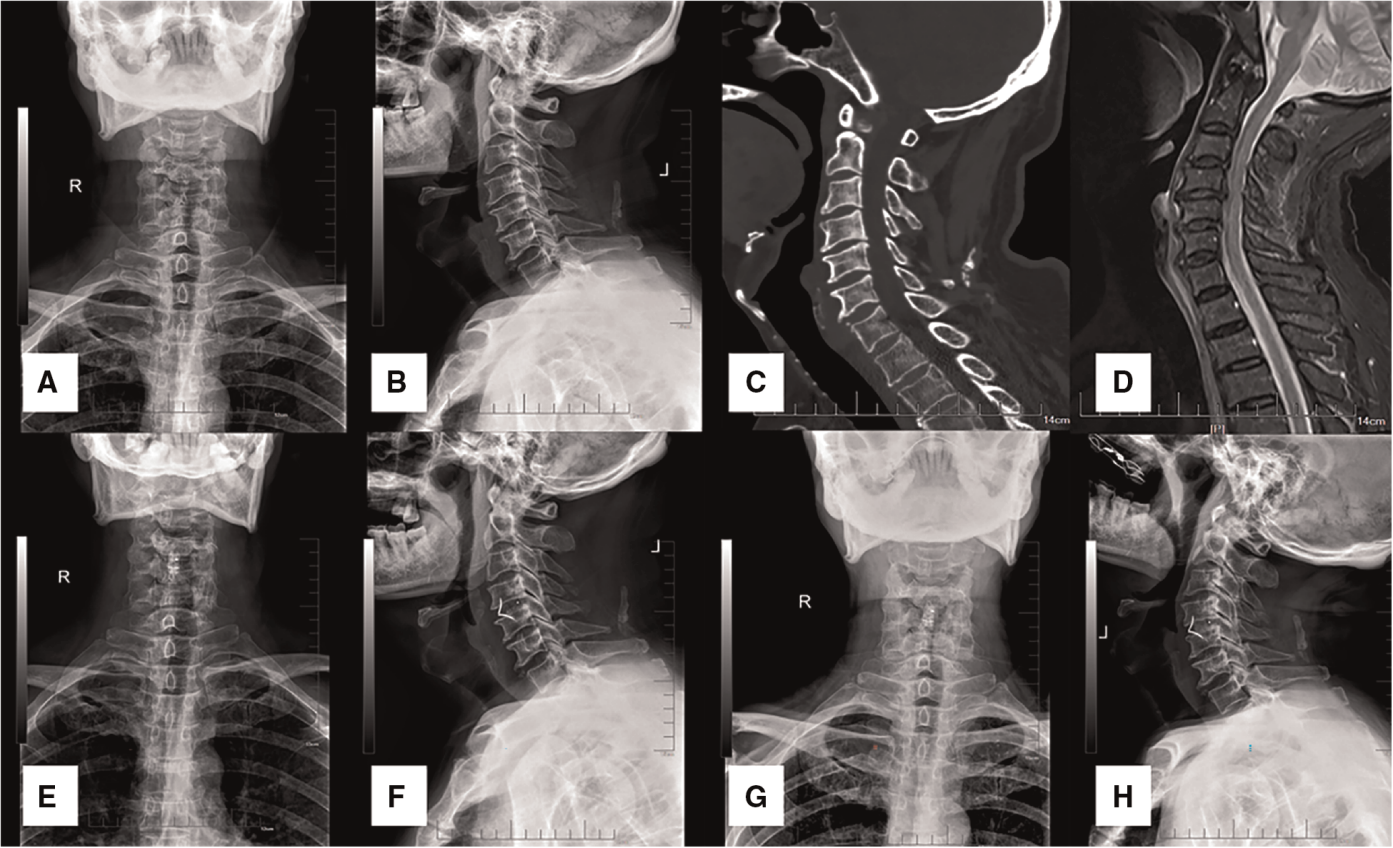
A 60-year-old male patient diagnosed with TCCS. (**A,B**) preoperative A-P and lateral x-ray showing the slight degenerative change of cervical spine with relative normal curvature, and conspicuous anterior osteophyte formation. (**C**) preoperative CT scan demonstrated no fracture or dislocation of the cervical spine. (**D**) preoperative MRI from short time inversion recovery (STIR) illustrated significant cervical spinal cord compression at C4–5 with spinal cord signal change at the same level, while evident prevertebral hyperintense and slight elevation of the ligament was also detected. (**E,F**) postoperative A-P and lateral x-ray at discharge indicate well positioned ROI-C device with normal CA and disc height. (**G,H**) the CA and disc height remained at final follow-up, and solid fusion was achieved.

**Figure 2:**
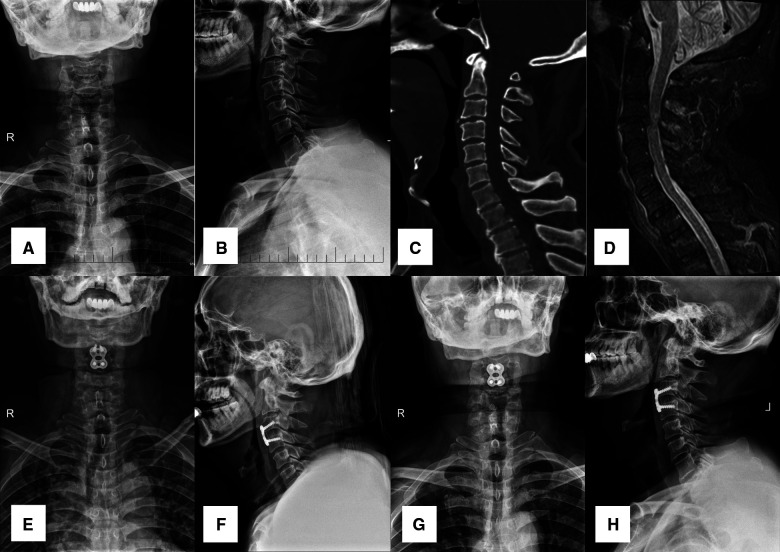
A 56-year-old male patient diagnosed with TCCS. (**A,B**) preoperative A-P and lateral X-ray images showing the slight degenerative change of cervical spine with relative normal curvature, and slight anterior osteophyte formation. (**C**) preoperative CT scan presented no fracture or dislocation of the cervical spine. (**D**) preoperative MRI from STIR displayed significant cervical spinal cord compression at C3-4 with spinal cord signal change at the same level, while distinct prevertebral hyperintense was also detected. (**E,F**) postoperative A-P and lateral X-ray images at discharge indicated APCS was in the appropriate site, and disc height was restored to a normal degree. (**G,H**) both CA and disc height were maintained at final follow-up.

All patients included underwent anteroposterior and lateral x-rays (Figures 1, 2). Cobb's method was applied to measure cervical angle (CA)which was the angle between the lower vertebral endplate of C2 and the upper endplate of C7. The intervertebral height was calculated as the mean value of the anterior, midline, and posterior distance between the inferior endplate of the cephalad vertebral body to the superior endplate of the caudal vertebral body of the operated segment. Fusion was considered as the absence of a radiolucent gap between the graft and the endplate, and evidence of continuous bridging trabecular bone at the fusion interface. Computed tomography (CT) would be performed if there was any controversy in the determination of fusion. Subsidence was assessed according to the criteria of device penetration into the endplates for more than 3 mm (14, 15). Adjacent level degeneration (ALD) was detected based on narrowing of intervertebral space and new osteophyte formation at adjacent interspace (9).

### Clinical outcome assessment

Patients' operative time and intraoperative blood loss were recorded. ASIA (American Spinal Injury Association) Impairment Scale was applied to assess neurological status, which consists of 5 grades each (From A to E). Furthermore, ASIA Impairment Scale was evaluated through motor scores and sensory scores. Japanese Orthopaedic Association (JOA) score and the neck disability index (NDI) score were also evaluated. Recovery rate (RR) of JOA was calculated as (postoperative JOA scores- preoperative JOA scores)/(17- preoperative JOA scores) × 100%. RR of JOA was interpreted as ≥75% (excellent), 50% to 74% (good), 25% to 49% (fair), and <25% (poor). Recovery rate of NDI was calculated as (preoperative NDI scores- postoperative NDI scores)/ (preoperative NDI scores) × 100%. Similarly, recovery rate of ASIA scores was calculated as (postoperative ASIA scores- preoperative ASIA scores)/ (full scores- preoperative ASIA scores) × 100%. Dysphagia-related symptoms were identified according to the system defined by Bazaz (12).

### Statistical analysis

Continuous variables were presented as mean ± standard deviation. The normal distribution of the continuous variable was tested by Kolmogorov-Smirnov test. Unpaired Student's *t*-test was used to analyze the 2 procedures. A paired sample *t*-test was applied to test data between preoperative and postoperative status. Chi-square test was used to assess categorical variables. A *P*-value less than 0.05 was considered to be statistically significant. SPSS 19.0 (SPSS Inc, Illinois, USA) was used for statistical analysis.

## Results

**Figure 3:**
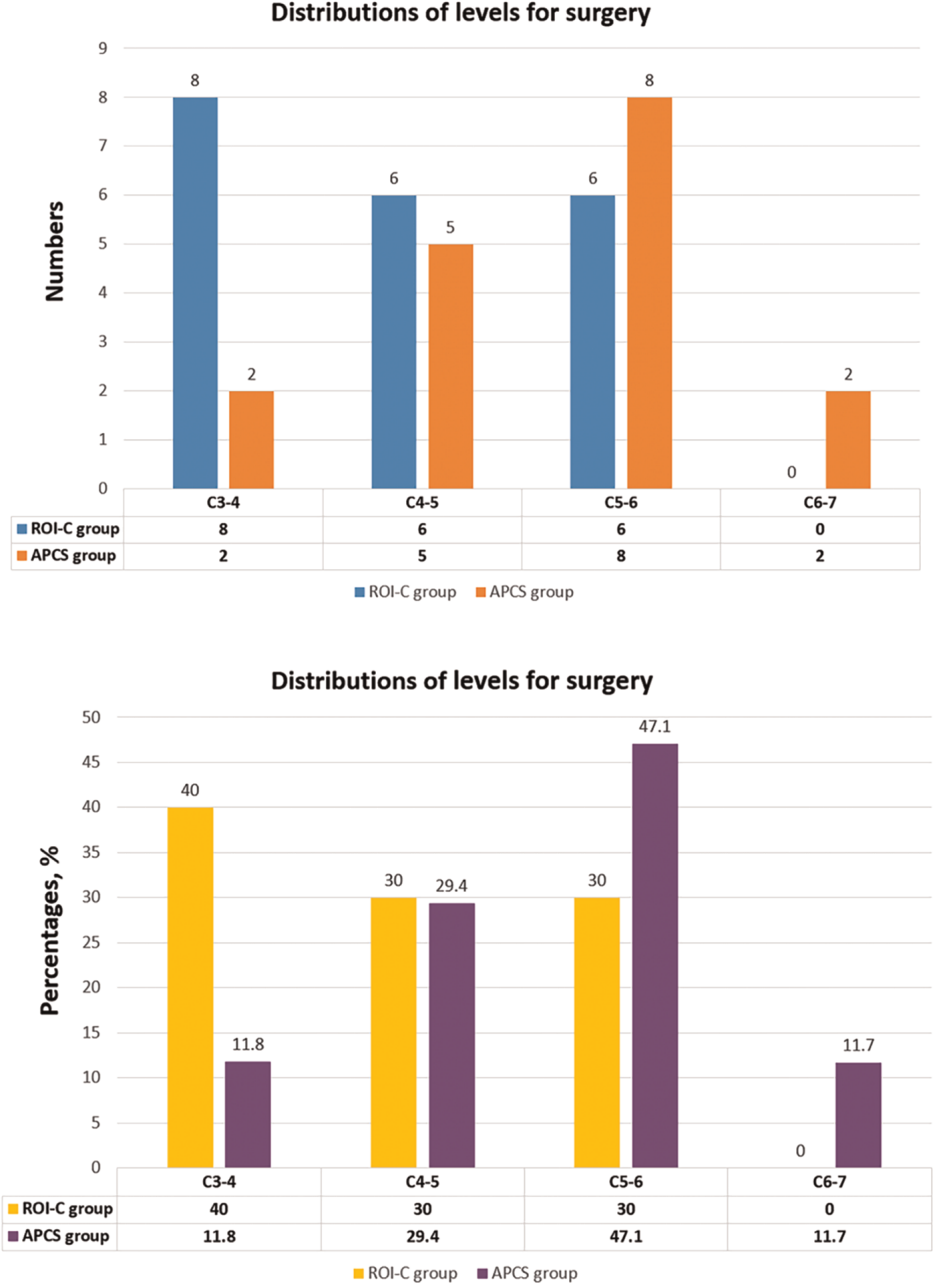
The distributions of levels for surgery in the ROI-C group and APCS group, exhibited as numbers and percentages.

The demographic data were revealed in Table 1. A total of 37 patients (30 males and7 females) with 37 levels achieved final follow-up. Twenty patients underwent 1-level ACDF with ROI-C device, and 17 patients received 1-level ACDF with APCS. The distribution of operated levels was presented in Figure 3. The mean age in the ROI-C group was 51.90 ± 9.64, and 50.00 ± 9.37 in the APCS group. The average follow-up time was 25.00 ± 7.99 months in the ROI-C group, and 21.29 ± 7.41 months in the APCS group. All devices were successfully implanted and anchored. ROI-C group had less operative time and less blood loss than the APCS group (89.40 ± 14.03 min vs. 110.29 ± 12.31 min, *P* < 0.05; 58.50 ± 7.72 ml vs. 93.53 ± 15.18 ml, *P* < 0.05).

### Clinical evaluation

The preoperative JOA and NDI scores did not significantly differ between the ROI-C and APCS groups. Meanwhile, no statistical difference between each postoperative time point and final follow-up. However, the JOA scores and NDI improved significantly from preoperative to postoperative time points (*P* < 0.05) and were maintained at final follow-up in each group. There was no statistical difference between the 2 groups regarding RR of JOA, NDI, total motor scores and sensory scores at final follow-up (*P* > 0.05) (Table 2).

**Table 2:** Clinical assessment of JOA, NDI, ASIA scores and radiographic measurement.

	ROI-C	APCS
JOA scores		
Admission	8.85 ± 0.81	8.82 ± 0.95
Discharge	13.55 ± 1.47*	13.35 ± 1.12*
Postoperative 3 months	14.45 ± 1.19*	14.35 ± 0.93*
Final follow-up	14.95 ± 0.99*	14.88 ± 0.78*
Recovery rate	75.14 ± 11.29	73.42 ± 8.15
NDI scores		
Admission	34.25 ± 4.28	33.82 ± 4.45
Discharge	14.65 ± 3.36*	15.65 ± 3.99*
Postoperative 3 months	12.90 ± 2.63*	13.65 ± 3.28*
Final follow-up	11.15 ± 1.95*	11.88 ± 2.57*
Recovery rate	67.48 ± 3.52	65.19 ± 3.83
ASIA scores		
Upper extremities motor scores		
Admission	21.05 ± 3.24	20.76 ± 3.17
Discharge	30.45 ± 3.39*	30.07 ± 4.07*
Postoperative 3 months	34.10 ± 3.21*	34.47 ± 4.09*
Final follow-up	38.10 ± 3.42*	37.65 ± 3.67*
Recovery rate	58.96 ± 10.99	57.25 ± 12.79
Lower extremities motor scores		
Admission	37.15 ± 2.09	36.59 ± 3.28
Discharge	41.10 ± 1.94*	39.94 ± 3.15*
Postoperative 3 months	42.80 ± 1.96*	41.94 ± 2.66*
Final follow-up	45.05 ± 1.82*	43.94 ± 2.01*
Recovery rate	61.97 ± 10.82*	54.75 ± 9.18*
Total motor scores		
Admission	58.20 ± 4.73	57.35 ± 5.50
Discharge	71.55 ± 4.49*	70.65 ± 5.97*
Postoperative 3 months	76.90 ± 4.29*	76.41 ± 5.21*
Final follow-up	83.15 ± 3.84*	81.58 ± 4.46*
Recovery rate	59.74 ± 7.87	56.23 ± 9.05
Difference of upper and lower extremities motor score		
Admission	16.10 ± 2.69	15.82 ± 3.38
Discharge	10.65 ± 3.23*	9.23 ± 4.18*
Postoperative 3 months	8.70 ± 3.15*	7.47 ± 4.53*
Final follow-up	6.95 ± 3.90*	6.29 ± 3.90*
Sensory scores		
Admission	79.15 ± 9.48	78.59 ± 7.90
Discharge	103.95 ± 14.26*	102.29 ± 12.49*
Postoperative 3 months	144.20 ± 14.78*	141.35 ± 12.44*
Final follow-up	147.65 ± 15.02*	144.88 ± 12.26*
Recovery rate	47.60 ± 7.72	45.74 ± 7.04
CA (degree, °)		
Admission	13.82 ± 2.58	13.86 ± 3.38
Discharge	17.57 ± 2.26*	17.71 ± 2.91*
Postoperative 3 months	17.16 ± 2.11*	17.46 ± 2.94*
Final follow-up	16.97 ± 2.07*	17.35 ± 2.93*
Disc height (mm)		
Admission	4.63 ± 0.50	4.43 ± 0.40
Discharge	6.16 ± 0.45*	6.24 ± 0.63*
Postoperative 3 months	5.96 ± 0.50*	6.13 ± 0.64*
Final follow-up	5.81 ± 0.50*	6.06 ± 0.61*

JOA, Japanese Orthopaedic Association; NDI, neck disability index; ASIA, American Spinal Injury Association; CA, cervical angle.

*Statistical significance achieved compared to preoperative value (*P* < 0.05).

ASIA Impairment Scale and grading results were demonstrated in Tables 2, 3. All patients included ranged from C to D at admission. The upper and lower extremities motor scores were analyzed separately, and the preoperative difference value was >10. Significant improvement was detected postoperatively and maintained at 3 months postoperatively and final follow-up time compared with preoperative values. No significant differences were detected between the two groups at each follow-up time.

**Table 3:** ASIA grading for neurological status.

	ROI-C (*n* = 20)	APCS (*n* = 17)
Admission		
A	0	0
B	0	0
C	5	4
D	15	13
E	0	0
Discharge		
A	0	0
B	0	0
C	2	2
D	12	13
E	4*^,^**	2*^,^**
Postoperative 3 months		
A	0	0
B	0	0
C	0	0
D	4	3
E	16*^,^**	14*^,^**
Final follow-up		
A	0	0
B	0	0
C	0	0
D	1	1
E	19*^,^**	16*^,^**

*Significant difference compared with the previous time point (*χ*^2^ test), *P* < 0.05.

**Significant difference compared with preoperative time point at admission (*χ*^2^ test), *P* < 0.05.

### Radiological assessment

The radiological outcomes were illustrated in Table 2. The preoperative CA and disc height of operated level did not vary between the 2 groups (*P* > 0.05). In both groups, the CA and disc height of operated level significantly increased postoperatively (*P* < 0.05) and maintained at 3 months postoperatively and in the final follow-up, respectively.

### Complications

Fusion was achieved in all patients. No deep infections, hematomas, bolt loosening, or breakage of anchoring clips, screws, or titanium plates were observed in both groups during the follow-up period. There were 2 patients complained of postoperative mild dysphagia (2/20, 10.0%) in the ROI-C group and 8/17 (47.1%) in the APCS group at discharge, which all recovered within 3 weeks. There was a statistical difference between the 2 groups as the ROI-C group had a lower incidence of dysphagia than the APCS group postoperatively (*P* < 0.05). Only 1 case of ALD was observed at final follow-up in the APCS group with no significant difference (*P* < 0.05). No subsidence was detected in both groups.

## Discussion

The distinctive clinical characteristic of TCCS distinguished from other types of SCIs is exhibited as disproportionate impairment of the upper limbs compared with the lower limbs in motor function damage. MH Pouw et al. (16) stratified TCCS through a quantitative and reproducible diagnostic criterion. Moreover, the subsequent study demonstrated a minimal difference of 10 ASIA motor score points between the upper and lower extremities, in favor of the lower extremities in TCCS patients (17, 18). This quantified criterion is consistent with the result of our study as all patients in both groups had great than 10 points differences between upper and lower extremities motor scores at admission, which made the selection of the targeted population more accurate. However, for injury at the lower cervical level (C7- T1), this difference was regarded to be too high. In addition, there was no patient included with spinal impairment below the level of C6–7 in our study, which is similar to the previous research (18).

Segment instability is an explicit indication for surgery, as increased range of motion at injured level of the cervical spine may lead to further compression of spinal cord. Assessment of discoligamentous complex is important in determining spinal stability. However, in patients with TCCS without cervical vertebral fracture or dislocation, the conventional radiographic assessment of stability has limited ability, because lateral radiographs in maximum extension are not recommended to avoid further spinal cord impairment (19). Although MRI findings cannot match all signs intraoperatively, it still has an acceptable sensitivity in detecting segment instability by inspecting anterior longitudinal ligament (ALL) disruption manifested as discontinuity of the hypointense band with prevertebral hyperintense or elevation of the ligament from adjacent structures. In the present study, all patients had prevertebral hyperintense signal preoperatively, and all confirmed appearing ALL rupture intraoperatively, indicating spinal instability.

ACDF can achieve direct decompression of spinal cord or nerve roots, reconstruction of cervical lordotic alignment, improvement of intervertebral height, solid fusion with smaller trauma, and less blood loss. Anterior decompression and intervertebral autologous bone grafting without fixation have been demonstrated to have an ideal incidence of fusion (20). However, it can result in graft displacement, subsidence, further damage to the nerve, kyphotic deformity change, and donor site complications (7). APCS can provide extra stability. However, it is connected with the following complications: plate and screw displacement, loosening, patient dysphagia, and future degeneration of adjacent discs (8, 9, 11, 21). Integrated with characters of both, ZPAC implant was invented with various designs. With no prevertebral occupation, ZPAC has been demonstrated with a relatively lower incidence of dysphagia than APCS, while similar fusion rate, JOA score improvement, and cervical lordosis were observed for single or multiple levels uses in contrast with APCS (8, 9, 14, 22). This was consistent with our findings as ROI-C device was related to a lower incidence of postoperative dysphagia, shorter operation time and less blood loss.

Several *in vitro* biomechanical studies were conducted with different versions of ZPAC. Scholz et al. (23) compared the locked version of ZPAC with APCS in 1-level ACDF, while Clavenna et al. (24) later compared the variable-angle version of ZPAC with APCS in 2 and 3-level ACDF. Their results were similar as no significant difference was detected in ROM during flexion-extension, lateral bending or axial rotation, although APCS displayed slightly better stability in flexion-extension, deducing equivalent stability of the 2 devices. Paik et al. (25) demonstrated that no difference was achieved in ROM between another sort of variable-angle screw version of ZPAC and APCS in 1-level application, but less stability was observed in ZPAC when operated in 2–3 levels. Their explanation was different designs of devices as screws of ZPAC did not provide lag compression or solid locking. The ROI-C device, integrated with 2 anchoring locking clips, enhances the stability of stand-alone cages (9, 10, 13). ROI-C has been proven to be an effective method for 1–2 level ACDF or even treating Hangman fracture (9, 10, 13). Interestingly, it remains obscure how much stability or ROM reduction is required for solid interbody fusion (26) as the weakness in flexion-extension of ZPAC did not influence the clinical results. In our study, all patients who underwent single-level ACDF with ROI-C showed significant improvement in JOA, NDI and ASIA scores, and fusion was achieved in all cases. These may be attributed to our cautious management: (1) optimal preparation of fusion surface without excessive damage to the bony endplate; (2) neck brace was used routinely postoperatively; (3) few osteoporotic patients were operated in this study, while it was usually encountered in cadaveric biomechanical researches.

Both CA and disc height significantly increased in ROI-C group in our study, which was consistent with previous studies treating cervical spondylotic myelopathy, indicating its good efficacy in restoring cervical alignment. However, during the postoperative 3 months of follow-up and later final follow-up, a slight loss of CA and disc height were observed without statistical significance. No subsidence was detected. Nevertheless, no clip breakage, displacement or loosening happened during each follow-up time. Apart from the advantages of the device, it may partly be due to the relatively young age and good bone quality of patients.

Plate thickness of APCS device is considered a primary factor that results in postoperative dysphagia as it may cause impingement or irritation to the ventral esophagus (21). ROI-C device is embedded in intervertebral space without protrusion at prevertebral space, which can reduce the incidence of dysphagia (8–10, 14, 27). However, intraoperative traction and mechanical stimulation of prevertebral tissues, postoperative local edema, hematoma and operation time also play roles in causing dysphagia after ACDF (27). ROI-C retrenches the operation time while ACPS has to consume more in preparing for plate installation, measurement and adjustment. Furthermore, different from the previous Zero-*P* device, no screw is applied in ROI-C devices (9, 10), which avoided various problems during screw insertion and further loosening. Fortunately, the dysphagia after ACDF is usually temporary, and most of them are relieved within 3 months postoperatively (8, 12, 27).

There exists a long-standing controversy about the optimal timing for surgery in patients with TCCS. Urgent surgery (≤24 h of injury) is recommended for acute instability of dislocation and progressive neurologic deficit, and it was associated with better recovery than delayed surgery or conservative treatment. However, the definition of early decompression is fuzzy as various studies set 24 h or 72 h as the time limit (3). Lenehan et al. (28) suggested that it is reasonable and safe to consider early surgical decompression in patients with a profound neurologic deficit (ASIA = C) and persistent spinal cord compression due to developmental cervical spinal canal stenosis without fracture or instability, while patients with a slight deficit (ASIA = D) could be treated with initial observation with surgery potentially at a later date depending on the extent and temporal profile of the patients' neurologic recovery. In our study, the average time before operation is beyond 72 h in both groups. The plausible explanations are indicated as follows: (1) a majority of patients included in the study are ASIA D, which opportunities could be given for observation before surgery according to the above viewpoint; (2) some patients were accompanied with underlying diseases such as associated slight injuries in brain and chest, which need careful consideration before surgery. Although relative late surgery time was observed in the current study, significant neurologic improvement was achieved at subsequent follow-up time.

The following limitations of this study were noted: (1) the retrospective character and small sample size; (2) MRI was not performed routinely in detecting ALD, which early degeneration may be neglected. Further large sample size, well-designed prospective randomized trials could be conducted comparing ROI-C and APCS devices in multilevel applications.

## Conclusion

Both ROI-C device and APCS demonstrated satisfactory clinical effects and safety in managing symptomatic single-level TCCS with underlying instability. Both techniques could improve and maintain cervical lordosis and disc height. ROI-C device was related to a lower incidence of postoperative dysphagia, shorter operation time, and less blood loss.

## Data Availability

The original contributions presented in the study are included in the article/Supplementary Material, further inquiries can be directed to the corresponding author/s.

## References

[1] Merriam WF Taylor TK Ruff SJ McPhail MJ. A reappraisal of acute traumatic central cord syndrome. J Bone Joint Surg Br. (1986) 68(5):708–13. 10.1302/0301-620X.68B5.3782229 3782229

[2] Schneider RC Cherry G Pantek H. The syndrome of acute central cervical spinal cord injury; with special reference to the mechanisms involved in hyperextension injuries of cervical spine. J Neurosurg. (1954) 11(6):546–77. 10.3171/jns.1954.11.6.0546 13222164

[3] Molliqaj G Payer M Schaller K Tessitore E. Acute traumatic central cord syndrome: a comprehensive review. Neurochirurgie. (2014) 60(1–2):5–11. 10.1016/j.neuchi.2013.12.002 24613283

[4] Wang B Liu H Wang H Zhou D. Segmental instability in cervical spondylotic myelopathy with severe disc degeneration. Spine (Phila Pa 1976). (2006) 31(12):1327–31. 10.1097/01.brs.0000218508.86258.d4 16721294

[5] Laporte C Laville C Lazennec JY Rolland E Ramare S Saillant G. Severe hyperflexion sprains of the lower cervical spine in adults. Clin Orthop Relat Res. (1999) (363):126–34. PMID: 10379314

[6] Lee YS Kim YB Park SW. Risk factors for postoperative subsidence of single-level anterior cervical discectomy and fusion: the significance of the preoperative cervical alignment. Spine (Phila Pa 1976). (2014) 39(16):1280–7. 10.1097/brs.0000000000000400 24827519

[7] Lee YS Kim YB Park SW. Does a zero-profile anchored cage offer additional stabilization as anterior cervical plate? Spine (Phila Pa 1976). (2015) 40(10):E563–70. 10.1097/brs.0000000000000864 25955093

[8] Wang ZD Zhu RF Yang HL Gan MF Zhang SK Shen MJ The application of a zero-profile implant in anterior cervical discectomy and fusion. J Clin Neurosci. (2014) 21(3):462–6. 10.1016/j.jocn.2013.05.019 24262773

[9] Wang Z Jiang W Li X Wang H Shi J Chen J The application of zero-profile anchored spacer in anterior cervical discectomy and fusion. Eur Spine J. (2015) 24(1):148–54. 10.1007/s00586-014-3628-9 25337859

[10] Grasso G Giambartino F Tomasello G Iacopino G. Anterior cervical discectomy and fusion with ROI-C peek cage: cervical alignment and patient outcomes. Eur Spine J. (2014) 23(Suppl 6):650–7. 10.1007/s00586-014-3553-y 25200146

[11] Fountas KN Kapsalaki EZ Nikolakakos LG Smisson HF Johnston KW Grigorian AA Anterior cervical discectomy and fusion associated complications. Spine (Phila Pa 1976). (2007) 32(21):2310–7. 10.1097/BRS.0b013e318154c57e 17906571

[12] Bazaz R Lee MJ Yoo JU. Incidence of dysphagia after anterior cervical spine surgery: a prospective study. Spine (Phila Pa 1976). (2002) 27(22):2453–8. 10.1097/01.brs.0000031407.52778.4b 12435974

[13] Cao G Meng C Zhang W Kong X. Operative strategy and clinical outcomes of ROI-C(TM) fusion device in the treatment of Hangman’s fracture. Int J Clin Exp Med. (2015) 8(10):18665–72. PMID: ; PMCID: 26770480 PMC4694380

[14] Njoku IJ Alimi M Leng LZ Shin BJ James AR Bhangoo S Anterior cervical discectomy and fusion with a zero-profile integrated plate and spacer device: a clinical and radiological study: clinical article. J Neurosurg Spine. (2014) 21(4):529–37. 10.3171/2014.6.spine12951 25105338

[15] Chen Y Wang X Lu X Yang L Yang H Yuan W Comparison of titanium and polyetheretherketone (PEEK) cages in the surgical treatment of multilevel cervical spondylotic myelopathy: a prospective, randomized, control study with over 7-year follow-up. Eur Spine J. (2013) 22(7):1539–46. 10.1007/s00586-013-2772-y 23568254 PMC3698331

[16] Pouw MH van Middendorp JJ van Kampen A Hirschfeld S Veth RPH EM-SCI study group Diagnostic criteria of traumatic central cord syndrome. Part 1: a systematic review of clinical descriptors and scores. Spinal Cord. (2010) 48(9):652–6. 10.1038/sc.2009.155 20048754

[17] van Middendorp JJ Pouw MH Hayes KC Williams R Chhabra HS Putz C Diagnostic criteria of traumatic central cord syndrome. Part 2: a questionnaire survey among spine specialists. Spinal Cord. (2010) 48(9):657–63. 10.1038/sc.2010.72 20585327

[18] Pouw MH van Middendorp JJ van Kampen A Curt A van de Meent H Hosman AJ. Diagnostic criteria of traumatic central cord syndrome. Part 3: descriptive analyses of neurological and functional outcomes in a prospective cohort of traumatic motor incomplete tetraplegics. Spinal Cord. (2011) 49(5):614–22. 10.1038/sc.2010.171 21151190

[19] Krappinger D Lindtner RA Zegg MJ Henninger B Kaser V Spicher A Spondylotic traumatic central cord syndrome: a hidden discoligamentous injury? Eur Spine J. (2019) 28(2):434–41. 10.1007/s00586-018-5796-5 30341627

[20] Jacobs W Willems PC Kruyt M van Limbeek J Anderson PG Pavlov P Systematic review of anterior interbody fusion techniques for single- and double-level cervical degenerative disc disease. Spine (Phila Pa 1976). (2011) 36(14):E950–60. 10.1097/BRS.0b013e31821cbba5 21522044

[21] Lee MJ Bazaz R Furey CG Yoo J. Influence of anterior cervical plate design on Dysphagia: a 2-year prospective longitudinal follow-up study. J Spinal Disord Tech. (2005) 18(5):406–9. 10.1097/01.bsd.0000177211.44960.71 16189451

[22] Vanek P Bradac O Delacy P Lacman J Benes V. Anterior interbody fusion of the cervical spine with Zero-P spacer: prospective comparative study-clinical and radiological results at a minimum 2 years after surgery. Spine (Phila Pa 1976). (2013) 38(13):E792–7. 10.1097/BRS.0b013e3182913400 23524869

[23] Scholz M Schnake KJ Pingel A Hoffmann R Kandziora F. A new zero-profile implant for stand-alone anterior cervical interbody fusion. Clin Orthop Relat Res. (2011) 469(3):666–73. 10.1007/s11999-010-1597-9 20882376 PMC3032850

[24] Clavenna AL Beutler WJ Gudipally M Moldavsky M Khalil S. The biomechanical stability of a novel spacer with integrated plate in contiguous two-level and three-level ACDF models: an in vitro cadaveric study. Spine J. (2012) 12(2):157–63. 10.1016/j.spinee.2012.01.011 22405617

[25] Paik H Kang DG Lehman RA Jr Cardoso MJ Gaume RE Ambati DV Do stand-alone interbody spacers with integrated screws provide adequate segmental stability for multilevel cervical arthrodesis? Spine J. (2014) 14(8):1740–7. 10.1016/j.spinee.2014.01.034 24462812

[26] Scholz M Schleicher P Pabst S Kandziora F. A zero-profile anchored spacer in multilevel cervical anterior interbody fusion: biomechanical comparison to established fixation techniques. Spine (Phila Pa 1976). (2015) 40(7):E375–80. 10.1097/brs.0000000000000768 25584947

[27] Barbagallo GM Romano D Certo F Milone P Albanese V. Zero-P: a new zero-profile cage-plate device for single and multilevel ACDF. A single institution series with four years maximum follow-up and review of the literature on zero-profile devices. Eur Spine J. (2013) 22(Suppl 6):S868–78. 10.1007/s00586-013-3005-0 24061968 PMC3830046

[28] Lenehan B Fisher CG Vaccaro A Fehlings M Aarabi B Dvorak MF. The urgency of surgical decompression in acute central cord injuries with spondylosis and without instability. Spine (Phila Pa 1976). (2010) 35(21 Suppl):S180–6. 10.1097/BRS.0b013e3181f32a44 20881460

